# Training at moderate altitude improves submaximal but not maximal performance-related parameters in elite rowers

**DOI:** 10.3389/fphys.2022.931325

**Published:** 2022-10-14

**Authors:** Hugo Cerda-Kohler, Danni Haichelis, Patricia Reuquén, Bianca Miarka, Mark Homer, Daniel Zapata-Gómez, Esteban Aedo-Muñoz

**Affiliations:** ^1^ Escuela de Ciencias del Deporte y Actividad Física, Facultad de Salud, Universidad Santo Tomás, Santiago, Chile; ^2^ Departamento de Educación Física, eporte y Recreación, Facultad de Artes y Educación Física, Universidad Metropolitana de Ciencias de la Educación, Santiago, Chile; ^3^ Laboratory of Psychophysiology and Performance in Sports and Combats, Postgraduate Program in Physical Education, School of Physical Education and Sport, Federal University of Rio de Janeiro, Rio de Janeiro, Brazil; ^4^ Unidad de Fisiología del Ejercicio, Centro de Innovación, Clínica MEDS, Santiago, Chile; ^5^ Unidad de Ciencias Aplicadas al Deporte, Instituto Nacional de Deportes, Santiago, Chile; ^6^ Escuela de Ciencias de la Actividad Física, el Deporte y la Salud, Universidad de Santiago de Chile, Santiago, Chile; ^7^ Laboratorio de Ciencias de la Actividad Física, Facultad de Medicina, Universidad de Chile, Santiago, Chile; ^8^ School of Human and Social Sciences, Buckinghamshire New University, Buckinghamshire, United Kingdom

**Keywords:** altitude training, endurance performance, maximal oxygen uptake, ventilatory thresholds, economy of rowing

## Abstract

Maximal oxygen consumption (V̇O_2max_), physiological thresholds, and hemoglobin mass are strong predictors of endurance performance. High values of V̇O_2max_, maximal aerobic power (MAP), and power output at anaerobic thresholds are key variables in elite rowers. Endurance athletes often use altitude training as a strategy to improve performance. However, no clear evidence exists that training at natural altitude enhances sea-level performance in elite rowers. This study aimed to evaluate the effect of altitude training on rowing-performance parameters at sea level. The study was conducted on eleven rowers (Six females, five males) from the Chilean National Team during a 3-week moderate altitude training (∼2,900 m. a.s.l.) under the live high-train high (LHTH) model. It included a rowing ergometer maximal incremental test and blood analysis (pre and post-altitude). Gas exchange analysis was performed to measure V̇O_2max_, ventilatory thresholds (VTs) and rowing economy/efficiency (ECR/GE%). LHTL training improves performance-related variables at sea level (V̇E_max_: 3.3% (95% CI, 1.2–5.5); hemoglobin concentration ([Hb]): 4.3% (95% CI, 1.7–6.9); hematocrit (%): 4.5% (95% CI, 0.9–8.2); RBC (red blood cells) count: 5.3% (95% CI, 2.3–8.2); power at VT2: 6.9% (95% CI, 1.7–12.1), V̇E_VT2_: 6.4% (95% CI, 0.4–12.4); power at VT1: 7.3% (95% CI, 1.3–13.3), V̇E_VT1_: 8.7% (95% CI, 1.6–15.8)) and economy/efficiency-related variables (ECR_VT2_: 5.3% (95% CI, −0.6 to −10.0); GE(%): 5.8% (95% CI, 0.8–10.7)). The LHTH training decreased breathing economy at MAP (−2.8% (95% CI, 0.1–5.6)), pVT2 (−9.3% (95% CI, −5.9 to −12.7)), and pVT1 (−9.3% (95% CI, −4.1 to −14.4)). Non-significant changes were found for V̇O_2max_ and MAP. This study describes the effects of a 3-week moderate altitude (LHTH training) on performance and economy/efficiency-related variables in elite rowers, suggesting that it is an excellent option to induce positive adaptations related to endurance performance.

## Introduction

In competitions such as the Olympic Games or World Cups, the differences between first (Gold) and fourth place (no medal) could be as small as ∼1.5%–2.0% for some sports such as rowing (Smith and Hopkins, 2011); therefore, the benefits that training can deliver, even seemingly trivial ones, can be quite substantial in elite sport (DeWeese et al., 2015). The regular rowing competition takes place on a 2,000 m course, with world best times varying from ∼5.5 to 7.0 min (depending on the boat type and weather conditions), an average power output of 450–550 W per rower (Mäestu et al., 2005), delivered *via* a high maximal oxygen uptake (V̇O_2max_), lactate threshold and rowing economy (Secher, 1993; Ingham et al., 2002). However, assessing physiological performance on-water is somewhat complicated because external factors such as wind, water currents, and temperature may influence results. Therefore, rowing ergometers have facilitated training and provided a controllable and repeatable equipment to assess rowing and physiological performance (Ingham et al., 2002; Mäestu et al., 2005; Silva et al., 2021).

Maximal oxygen uptake, lactate/ventilatory thresholds, and efficiency/economy are strong predictors (key variables) of endurance performance (van der Zwaard et al., 2021), where cardiac output, hemoglobin mass/concentration, and skeletal muscle characteristics elucidate these physiological determinants (Joyner and Coyle, 2008; Lundby and Robach, 2015; van der Zwaard et al., 2021). An exceptional V̇O_2max_ is mandatory for elite endurance athletes (Lundby and Robach, 2015), representing an extraordinary maximal cardiac output and a high O_2_-carrying capacity, which increases oxygen delivery to the exercising muscles. This is represented by a close relationship between V̇O_2max_ and hemoglobin mass/concentration in elite athletes (Lundby and Robach, 2015). In rowing, research suggests that a high absolute V̇O_2max_ (values of 7 L/min in males and 5.5 L/min in females) (van der Zwaard et al., 2021) maximal aerobic power (MAP) and power output at anaerobic thresholds are key performance variables, the latter being one of the most relevant to improving performance parameters in highly-trained rowers (Steinacker, 1993; Ingham et al., 2002).

Endurance athletes who live at sea level often use altitude training as a strategy to improve performance (Burtscher et al., 2018). Today, evidence suggests that altitude training may benefit some athletes, and one of the accepted underlying mechanisms is an increase in hemoglobin mass/concentration and thus O_2_-carrying capacity (Levine and Stray-Gundersen, 1997; Robach and Lundby, 2012). In addition, adaptations induced in skeletal muscle could contribute to improving the whole-body exercise economy associated with altitude training (Gore et al., 2001). The basis for altitude training is the independent and combined physiological processes of acclimatization to chronic hypoxia and those derived from the additional stress imposed by training in a hypoxic environment (Rodríguez et al., 2015). Besides, recent publications show that the effectiveness of altitude training depends on many variables such as the time and magnitude of altitude, showing that the hematological response to altitude training depends on the hypoxic (at least 250 h) at a moderate altitude (Płoszczyca et al., 2018). However, no clear evidence exists that training at moderate natural altitude enhances sea-level performance in elite rowers.

Classic altitude training refers to the “live high-train high” (LHTH) model, which consists of living and training for weeks at moderate-to-high natural altitude. In addition to this model, other protocols of natural or artificial hypoxia as live-high train-low (LHTL), artificial LHTL with daily exposure to long (8–18 h) continuous, brief (1.5 to 5 h) continuous or brief (<1.5 h) intermittent periods of hypoxia, and artificial live-low train-high (LLTH), among others (Bonetti and Hopkins, 2009; Girard et al., 2017), has been used by endurance athletes in pursuit of performance enhancement after returning to sea level. Some evidence showed an increase in V̇O_2max_ with LHTH in sub-elite athletes, a possible reduction in elite athletes, and unclear effects with other protocols. Besides, enhancement of MAP in controlled studies of sub-elite athletes occurs with natural LHTL but is unclear with other protocols (Bonetti and Hopkins, 2009). Regarding hematological parameters, hemoglobin parameters demonstrated a likely moderate increase after LHTH training (Bonetti and Hopkins, 2009). Few studies have shown the effects of altitude training on performance in rowers, examining peripheral effects such as cutaneous microcirculation over four to 8 weeks of training (Meng et al., 2019; Meng et al., 2021). However, the evidence regarding the effect of LHTH training on V̇O_2max_, lactate/ventilatory thresholds, and exercise economy/efficiency in elite rowers is lacking.

Finally, it is proposed that the lack of improved performance in some key endurance variables with LHTH training could be related to managing training intensities in a hypoxic environment (Lundby and Robach, 2015). Maximal oxygen uptake decreases with increasing altitude, with ∼10% of the sea-level V̇O_2max_ value lost for every 1,000 m, starting at about 1,500 m; however, at any given altitude, the V̇O_2max_ decrement varies significantly between individuals, with the majority of the variance explained by the sea-level value (Brutsaert, 2008). The latter highlights the importance of individualizing training loads according to individual capacities assessed at altitude.

This study aimed to evaluate the effect of 3-week moderate altitude training (∼2,900 m. a.s.l.) on parameters related to rowing performance at sea level. It is hypothesized that altitude training will improve physiological thresholds and economy/efficiency-related variables in elite rowers.

## Material and methods

### Subjects

Eleven (Two Juniors, nine Seniors) heavyweight scull rowers, six females (V̇O_2max_: 3.9 ± 0.31 L/min; 58.2 ± 4.5 ml/kg/min), and five males (V̇O_2max_: 5.5 ± 0.31 L/min; 66.4 ± 3.7 ml/kg/min) participated in the study. The subject’s characteristics are presented in Table 1. All athletes were selected to the 2017 Chilean Rowing Team and categorized as “medal potential” for the Lima 2019 Pan American Games. This was the first experience in altitude training for all athletes. Inclusion criteria were: (i) being affiliated with the International Rowing Federation; (ii) having at least 5 years of training experience; (iii) having at least 18 years old; and (iv) having a minimum of 95% percentage of LHTH training frequency during the intervention. Exclusion criteria were (i) presence of osteoarticular injuries (*i.e.* injuries in bone tissues and joints): or muscle lesions during the study; (ii) could not complete the LHTL sessions and tests proposed by this research (iii) use of ergogenic substances (*i.e*., supplements); which could enhance physical performance or change V̇O_2max_ values (*e.g*., caffeine, creatine, taurine, and others), and; (iv) having participated in LHTH camp or hypobaric environment before the experiment. To our knowledge, the participants did not consume any dietary supplements or medication at the moment of the study.

**TABLE 1:** Basal characteristics before altitude training and water intake and hydration status during altitude.

	Females	Males
Demography/Anthropometry pre-altitude		
Age (years)	19.2 ± 1.0	22.2 ± 5.0
Height (cm)	172.4 ± 3.4	182.6 ± 5.3
Weight (kg)	66.3 ± 4.0	82.9 ± 7.6
Mean water intake and hydration values during altitude		
Water intake/day (L)	2.8 ± 0.2	4.6 ± 0.6
U.D. (g/ml)	1.018 ± 1.2	1.016 ± 1.9

U.D.: urine density.

The present study used pre-altitude data as a control moment to compare the effect of LHTH training. Since our research recruited all athletes of the national team, no other independent control group with the same level could be paired at the research moment to compare sea-level rowing training effects. The same group was in continuous rowing training under normoxic conditions at the pre-altitude moment. All athletes had state medical insurance and received information about the study objectives and the methodological procedures. They were fully informed of any risks and discomforts associated with these experiments before giving their informed consent to participate. Subsequently, participants signed the informed consent form. The study conformed with the code of Ethics of the World Medical Association (Declaration of Helsinki). In addition, the present research complies with the requirements of the international STROBE checklist (Vandenbroucke et al., 2014).

## Study design

### Testing procedures

**FIGURE 1:**
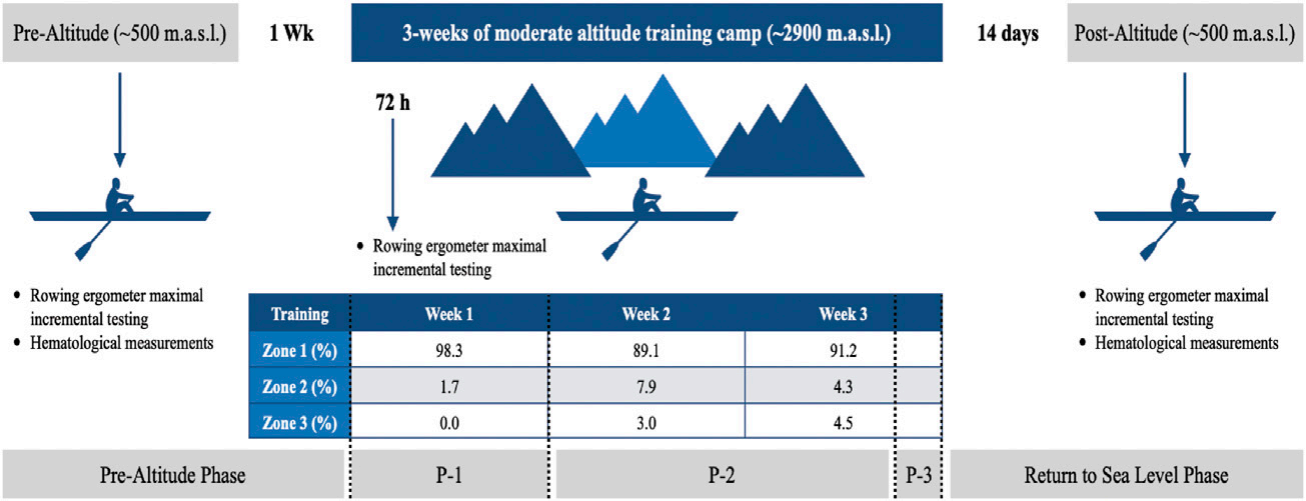
Schematic timeline of study design. The athletes were measured pre-intervention (pre-altitude) and 14 days after returning to sea level (post-altitude). m. a.s.l. meters above sea level; Wk: week. Zone 1: performed below the first ventilatory threshold (pVT1); Zone 2: performed between the first and second ventilatory thresholds (pVT1 and pVT2); Zone 3: performed above the second ventilatory threshold (pVT2). These zones were obtained in a rowing ergometer maximal incremental testing conducted 72 h after arriving at altitude. P-1: acclimatization phase; P-2: primary training phase; P-3: recovery and preparation for return to sea level phase.

Subjects arrived at the laboratory between 10:00 a.m. and 12:00 p.m., having fasted for at least 2 h and refrained from training for 48 h. All tests were performed in a temperature-controlled laboratory (18°C to 23°C, relative humidity <70%). Anthropometric, hematological measurements, and a rowing ergometer maximal incremental testing were conducted 1 week before and 14 days after the moderate altitude training (Rodríguez et al., 2015) (Figure 1). To adjust aerobic training zones (Stöggl and Sperlich, 2015) according to the new environment condition, a new rowing ergometer maximal incremental testing was conducted 72 h after arriving at the moderate altitude training (see Figure 1 for training intensity distribution).

### Maximal oxygen uptake, ventilatory thresholds, and economy/efficiency of rowing

After a 10-min warm-up at 100 W, the participants performed a maximal incremental test on a row-ergometer (L112) Concept 2, Model D, Nottingham, United Kingdom) according to the criteria previously described (Bentley et al., 2007). An initial workload of 100 W (women) and 150 W (men) was used, with increments of 20 W (women) and 30 W (men) every 1 min until exhaustion. Gas exchange was recorded continuously with a portable breath-to-breath gas analyzer (Cortex Metamax 3B, Leipzig, Germany). The analyzer was calibrated according to the manufacturer’s instructions before each trial. Pulmonary ventilation (V̇E), oxygen uptake (V̇O_2_), expired carbon dioxide (V̇CO_2_), and respiratory exchange ratio (RER) were averaged over 10 s in the mixing chamber mode, with the highest 15 s value used in the analysis. V̇O_2max_ was determined according to previously established criteria (Howley et al., 1995): (i) plateau in V̇O_2_ (*i.e*., increase <150 ml/min), (ii) RER >1.1, (iii) ≥90% of theoretical maximal heart rate, and (iv) blood lactate levels >8.0 mmol/L. The V̇O_2max_ was expressed as absolute values (L/min) and relative to body mass (ml/kg/min). Ventilatory threshold 1 (VT1) and ventilatory threshold 2 (VT2) were identified according to the following criteria (Cerezuela-Espejo et al., 2018):

- VT1 (*i.e.*, first physiological threshold): the intensity that causes the first rise in the ventilatory equivalent of oxygen (V̇E/VO_2_) without a concurrent rise in the ventilatory equivalent of carbon dioxide (V̇E/V̇CO_2_).
- VT2 (*i.e.*, second physiological threshold): the intensity that causes a rise in V̇E/V̇O_2_ and V̇E/V̇CO_2_ and a fall in end-tidal CO_2_ (PETCO_2_).

Mean RER values at pVT1 and pVT2 were 0.89 ± 0.018 and 0.96 ± 0.016, respectively. Rowing economy, calculated as the energy cost of rowing (ECR), was determined as the total V̇O_2_ equivalent (mLO_2_/min) divided by the power generated (W) at ventilatory thresholds intensities:

$$ECR\;\left(mLO_{2}/W\right)=\frac{Oxygen\;consumption}{power\;output}$$

As a complement, gross rowing efficiency (GE%) was calculated at pVT2 as previously described (Bourdin et al., 2004).

### Hematological measurements

An automated hematology analyzer Sysmex XN-1000 (Sysmex Corporation, Kobe, Japan), performed a complete blood count using whole venous blood according to the manufacturer’s instructions. Hematocrit (%), the concentration of hemoglobin ([Hb]; g/dL), and red blood cell (RBC; cells/mcL) count were measured.

#### Moderate altitude training camp

The 3-week moderate (Bärtsch and Saltin, 2008) altitude camp under the LHTH model took place in summer at the Portillo ski center, at an altitude of ∼2,900 m in the Andes Mountains. Altitude training was carried out in March 2017, with mean temperatures of 5°C and 17°C. The facilities had a 5-star Hotel with all the required amenities and a gym with free weights necessary to facilitate strength training (3 times per week). In addition, the facilities have a natural lagoon (Laguna del Inca, located 20 m from the hotel, with dimensions of 3,500 m long and 1,000 m wide, fully protected from the wind by the mountains, allowing training with specific competition boats. The rowers carried out two daily training sessions (see Figure 1 for intensity training distribution), the AM session in their specific boats and the PM in a row-ergometer or gym for strength work. Previously and during the moderate altitude camp, nutritional aspects were controlled by the nutrition team of the Applied Sports Science Unit from the High-Performance Center of Chile to maintain an adequate quality and quantity of nutrients and water according to the environment and the individual energy requirements (see Table 1 for water intake and hydration status during altitude training). Also, normal ferritin levels prior to altitude were confirmed (serum ferritin >35 ng/ml for females and >50 ng/ml for males) (Girard et al., 2013).

### Statistical analysis

All data were expressed as mean ± standard deviation (SD) or 95% confidence interval (CI). Data normality was initially confirmed by Shapiro–Wilk tests. Paired-sample t tests were used to test the null hypothesis stating no difference between pre and post values. The chances that change in performance or physiological variables were substantial (*i.e*., greater than the smallest worthwhile change, SWC), similar to or smaller than another time point was calculated. SWC was calculated as 0.2 multiplied by the between-subject standard deviation, based on Cohen’s d principle (Buchheit 2016a). Thresholds values for standardized changes/differences in the changes were >0.2 (small), >0.6 (moderate), >1.2 (large) and >2 (very large) (Lacome et al., 2020). We also used changes as a factor of variable-specific smallest worthwhile differences, where changes of 1×, 3×, 6×, and 10× SWC can be considered small, moderate, large, and very large (Buchheit, 2016b). This approach allows us to deliver the message for coaches and athletes as simple as “the effect is x times greater than the smallest worthwhile change.” If the 90% CL overlapped positive and negative values, the magnitude was unclear; otherwise, that magnitude was the observed magnitude. Statistical analysis was performed using STATA 13.0 (StataCorp, College Station, TX, United States). Statistical significance was set at *p* < 0.05.

## Results

Seventy-two hours after arriving at the moderate altitude training camp MAP, pVT2 and pVT1 decreased -14.6% (95% CI, −10.8 to −18.4), −9.5% (95% CI, −5.9 to −13.0), and −14.6% (95% CI, −9.2 to −20.0), respectively (MAP: 309 ± 60 W; power at VT2: 244 ± 55 W; power at VT1: 191 ± 45 W). Also, LHTH training period induced a substantial decrease in body weight [−1.1 kg, 95% CL (−0.3; −2.0), SWC-Factor = -4.5, 95% CL (−1.2; −7.9; *p* = 0.013), affecting the relative values of performance and economy-related variables. Basal values, the difference between pre and post, and effect size are shown in Table 2.

**TABLE 2:** Effects of a 3-week moderate altitude training camp on performance and economy/efficiency-related variables.

Variable	Pre	Post	ES [95% CI]	Rating	*p*-Value
Performance-related variables					
V̇O_2_max_abs_ (L/min)	4.59 ± 0.90	4.61 ± 0.97	0.16 [0.74, −0.42]	Unclear	0.587
V̇O_2_max_rel_ (ml/min/kg)	61.96 ± 5.82	63.17 ± 6.06	0.45 [1.07, −0.15]	Unclear	0.139
MAP (W)	362 ± 75	365 ± 78	0.23 [0.81, −0.35]	Unclear	0.432
V̇E_max_ (L/min)	160.9 ± 38.6	166.3 ± 40.0	0.95 [1.70, 0.27]	Moderate	0.007
[Hb] (gr/dL)	14.0 ± 1.1	14.6 ± 1.1	1.01 [1.78, 0.31]	Moderate	0.005
Hematocrit (%)	42.1 ± 3.4	44.0 ± 3.1	0.76 [1.46, 0.12]	Moderate	0.021
RBC (cells/mcL)	4.7 ± 0.4	4.9 ± 0.4	1.11 [1.91, 0.39]	Moderate	0.003
pVT2 (W)	269 ± 49	288 ± 58	0.83 [1.54, 0.17]	Moderate	0.014
pVT2 (%)	74.9 ± 5.8	79.2 ± 4.7	0.79 [1.49, 0.14]	Moderate	0.018
V̇O_2abs_ at pVT2 (L/min)	3.95 ± 0.78	3.99 ± 0.81	0.17 [0.75, −0.40]	Unclear	0.557
V̇E at VT2 (as % V̇E_max_)	71.5 ± 9.2	76.1 ± 6.7	0.66 [1.33, 0.04]	Moderate	0.039
pVT1 (W)	224 ± 46	240 ± 51	0.75 [1.44, 0.11]	Moderate	0.023
pVT1 (%)	62.3 ± 6.6	66.0 ± 4.0	0.76 [1.45, 0.12]	Moderate	0.021
V̇O_2abs_ at VT1 (L/min)	3.44 ± 0.79	3.53 ± 0.72	0.32 [0.92, −0.26]	Unclear	0.272
V̇E VT1 (as % V̇Emax)	57.7 ± 7.9	62.7 ± 4.5	0.76 [1.45, 0.11]	Moderate	0.022
Economy-related variables					
V̇E/V̇O_2_max	34.9 ± 2.6	35.9 ± 1.8	0.65 [1.31, 0.02]	Moderate	0.042
V̇E/V̇O_2_ VT2	28.7 ± 2.3	31.4 ± 1.9	1.71 [2.75, 0.82]	Large	<0.001
V̇E/V̇O_2_ VT1	26.8 ± 2.6	29.2 ± 1.3	1.12 [1.92, 0.39]	Moderate	0.003
ECR max (mLO_2_*W)	12.7 ± 0.6	12.7 ± 0.7	−0.05 [0.52, −0.62]	Unclear	0.865
ECR VT2 (mLO_2_*W)	14.7 ± 0.8	13.9 ± 0.9	−0.69 [−0.06, −1.37]	Moderate	0.032
ECR VT1 (mLO_2_*W)	15.3 ± 1.2	14.8 ± 0.9	−0.50 [0.10, −1.13]	Unclear	0.102
GE (%)	19.7 ± 1.0	20.9 ± 1.4	0.72 [1.40, 0.08]	Moderate	0.027

ES: effect size based on Cohen’s d; CI: confidence interval; MAP: maximal aerobic power; Hb: hemoglobin; RBC: red blood cell; pVT2: power at ventilatory threshold 2; pVT1: power at ventilatory threshold 1; V̇E: pulmonary ventilation; ECR: energy cost of rowing; GE: gross efficiency. Thresholds values for standardized changes/differences in the changes are >0.2 (small), >0.6 (moderate), >1.2 (large) and >2 (very large).

### Performance-related variables

#### Maximal values

**FIGURE 2:**
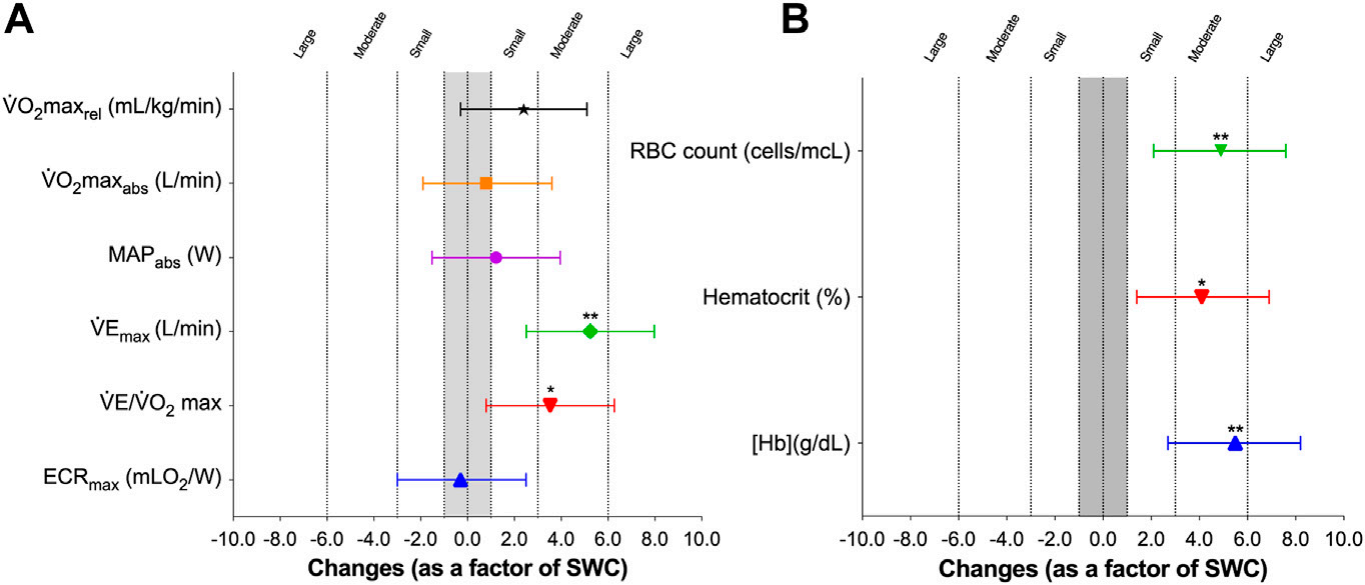
Effects of altitude training on maximal values **(A)** and hematological parameters **(B)** in elite rowers. SWC: smallest worthwhile change. Differences were calculated as a factor of variable-specific smallest worthwhile differences, where changes of 1×, 3×, 6×, and 10× SWC are considered small, moderate, large, and very large. Symbols and whiskers represent mean ± SD.

Figure 2A shows that LHTH induced an unclear effect on absolute V̇O_2max_ [0.03 L/min, 95% CL (−0.08; 0.13), SWC-Factor = 0.8, 95% CL (−2.5; 4.2; *p* = 0.587), relative V̇O_2max_ [1.21 ml/kg/min, 95% CL (−0.47; 2.90), SWC-Factor = 2.4, 95% CL (−0.9; 5.8; *p* = 0.139) and MAP [2.7 W, 95% CL (−4.7; 10.1), SWC-Factor = 1.2, 95% CL (−2.1; 4.6; *p* = 0.432), and a moderate increase on V̇E_max_ [5.32 L/min, 95% CL (1.85; 8.78), SWC-Factor = 5.2, 95% CL (1.8; 8.5; *p* = 0.007).

#### Hematological values

Regarding hematological variables, the LHTH induced a moderate increase (Figure 2B) on [Hb] [0.60 g/dl, 95% CL (0.23; 0.97), SWC-Factor = 5.5, 95% CL (2.1; 8.8; *p* = 0.005), hematocrit [1.91%, 95% CL (0.36; 3.46), SWC-Factor = 4.1, 95% CL (0.8; 7.5; *p* = 0.021), and a large increase in RBC count [0.25 cells/mcL, 95% CL (0.11; 0.39), SWC-Factor = 6.0, 95% CL (2.7; 9.4; *p* = 0.003).

#### Sub-maximal values

**FIGURE 3:**
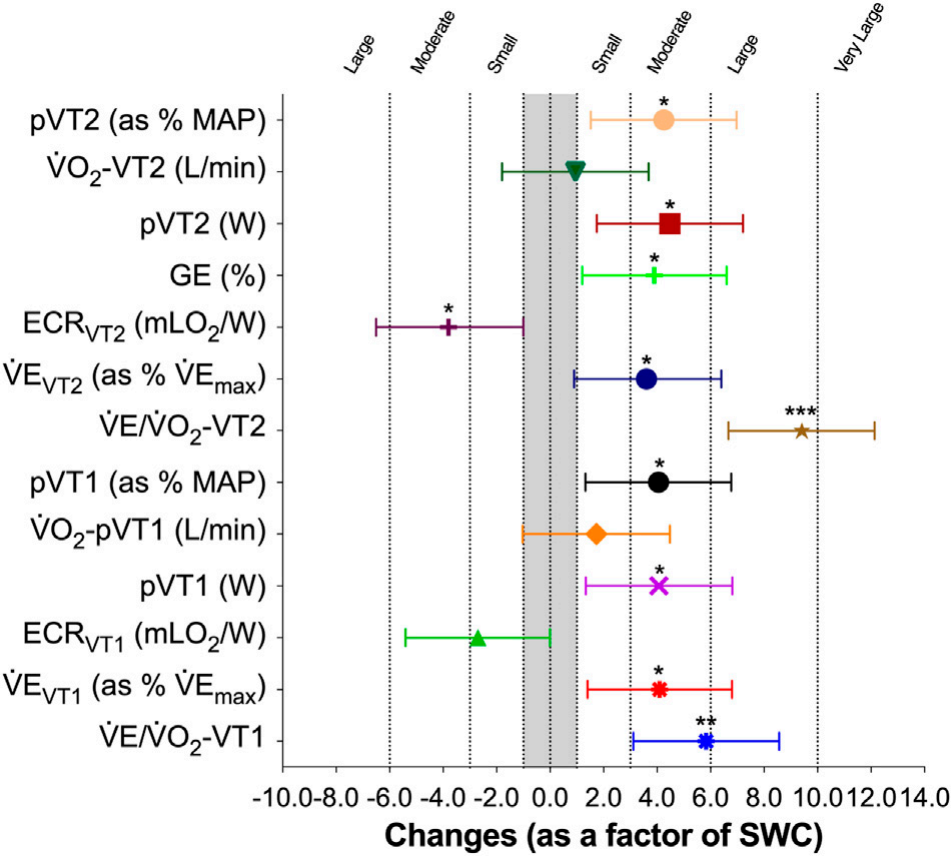
Effects of altitude training camp on performance, physiology-related economy/efficiency, and performance-related economy/efficiency in elite rowers. SWC: smallest worthwhile change. Differences were calculated as a factor of variable-specific smallest worthwhile differences, where changes of 1×, 3×, 6×, and 10× SWC are considered small, moderate, large, and very large. Symbols and whiskers represent mean ± SD.

The LHTH induced a moderate increase on power output at VT2 [18.6 W, 95% CL (4.7; 32.6), SWC-Factor = 4.5, 90% CL (1.1; 7.9; *p* = 0.014)], on power output at VT2 as a percentage of MAP [4.3%, 95% CL (0.9; 7.7)], SWC-Factor = 4.3, 90% CL (0.9; 7.6; *p* = 0.018) and V̇E at VT2 as a percentage of V̇E_max_ [4.6%, 95% CL (0.3; 8.8)], SWC-Factor = 3.6, 95% CL (0.2; 6.9; *p* = 0.039) (Figure 3). There was an unclear effect on V̇O_2_ at VT2 [0.04 L/min, 95% CL (−0.10; 0.18)], SWC-Factor = 0.9, 95% CL (−2.4; 4.3; *p* = 0.557).

Regarding variables associated to VT1 (Figure 3), there was a moderate increase on power output at VT1 [16.4 W, 95% CL (2.8; 29.9), SWC-Factor = 4.1, 95% CL (0.7; 7.4; *p* = 0.023)], on power output at VT1 as a percentage of MAP [3.8%, 95% CL (0.7; 6.8), SWC-Factor = 4.1, 95% CL (0.8; 7.5; *p* = 0.021)], and V̇E at VT1 as a percentage of V̇E_max_ [5.0%, 95% CL (0.9; 9.1), SWC-Factor = 4.1, 95% CL (0.7; 7.5; *p* = 0.022)]. No changes were observed on V̇O_2_ at VT1 [0.09 L/min, 95% CL (−0.08; 0.27), SWC-Factor = 1.8, 95% CL (−1.6; 5.2; *p* = 0.272)].

### Economy/efficiency -related variables

#### Physiology-related economy/efficiency

After the altitude training camp, there was a moderate increase in maximal ventilatory equivalents for oxygen (V̇E/V̇O_2_) at MAP [0.99, 95% CL (0.04; 1.94), SWC-Factor = 3.5, 95% CL (0.1; 6.9; *p* = 0.042)]. Regarding submaximal values, there was a decrease in breathing economy observed through a large increase in the ventilatory equivalents for oxygen (V̇E/V̇O_2_) at VT2 [2.67, 95% CL (1.70; 3.63), SWC-Factor = 9.3, 95% CL (5.9; 12.7; *p* = 0.000)], and a large increase in the ventilatory equivalents for oxygen (V̇E/V̇O_2_) at VT1 [2.48, 95% CL (1.10; 3.86), SWC-Factor = 6.0, 95% CL (2.7; 9.4; *p* = 0.003)], and (Figure 3).

### Performance-related economy/efficiency

Finally, there was un unclear effect of altitude training camp in the energy cost of rowing at MAP [−0.03 mLO_2_/W, 95% CL (−0.41; 0.35), SWC-Factor = −0.3, 95% CL (−3.6; 3.1; *p* = 0.865)], a moderate decrease at VT2 [−0.77 mLO_2_/W, 95% CL (−0.08; −1.47), SWC-Factor = −3.8, 95% CL (−0.4; −7.1; *p* = 0.032)], and an unclear effect at VT1 [−0.58 mLO_2_/W, 95% CL (−1.30; 0.14), SWC-Factor = −2.7, 95% CL (−6.1; 0.6; *p* = 0.102)] (Figure 3).

## Discussion

To our knowledge, this study is the first to describe the effect of a 3-week moderate altitude (∼2,900 m. a.s.l.) camp under the LHTH model on (i) performance-related variables and (ii) economy/efficiency-related variables in elite rowers. The main findings are as follows:

LHTH training improves performance-related variables (V̇E_max_: 3.3% (95% CI, 1.2–5.5) [Hb]: 4.3% (95% CI, 1.7–6.9); hematocrit: 4.5% (95% CI, 0.9–8.2); RBC count: 5.3% (95% CI, 2.3–8.2); pVT2: 6.9% (95% CI, 1.7–12.1), V̇E_VT2_: 6.4% (95% CI, 0.4–12.4); pVT1: 7.3% (95% CI, 1.3–13.3), V̇E_VT1_: 8.7% (95% CI, 1.6–15.8); and economy/efficiency-related variables (ECR_VT2_: 5.3% (95% CI, −0.6 to −10.0); GE (%): 5.8% (95% CI, 0.8–10.7)). Nevertheless, LHTH training also induces a decrease in breathing economy at MAP (−2.8% (95% CI, −0.1 to −5.6)), VT2 (−9.3% (95% CI, −5.9 to −12.7)), and VT1 (-9.3% (95% CI, −4.1 to −14.4)).

Our study aimed to describe the effect of a 3-week moderate altitude training (∼2,900 m. a.s.l.) camp under the LHTH model on rowing performance-related variables after returning to sea level. According to Millet et al. (Millet et al., 2010) the returning to sea level has three phases: (i) a positive phase observed during the first 2–4 days, but not in all athletes, (ii) a phase of progressive re-establishment of sea-level training volume and intensity, where the probability of good performance is reduced, and (iii) 15–21 days after return to sea level, a third phase characterized by a plateau in fitness. The optimal delay for competition is during this third phase, although some athletes reach their peak performance during the first phase. Unfortunately, published observations and the results of interventions examining the training practice of elite athletes are rare. In addition, limitations, such as the lack of control groups (as evident in this study), make the potential added value of such interventions challenging to assess.

Maximal oxygen uptake, MAP, power at lactate/ventilatory thresholds, and efficiency/economy are critical variables of endurance performance (van der Zwaard et al., 2021), whereas variables such as hemoglobin mass/concentration and skeletal muscle characteristics explain these physiological determinants (Joyner and Coyle, 2008; Lundby and Robach, 2015; van der Zwaard et al., 2021). Thus, the V̇O_2max_ and lactate/ventilatory thresholds interact to determine how long an athlete can sustain a given rate of aerobic and anaerobic metabolism, whereas efficiency determines the velocity or power that can be achieved with a given amount of energy consumption (Joyner and Coyle, 2008; Lundby and Robach, 2015; van der Zwaard et al., 2021). Our results showed that the LHTH training induces no changes in V̇O_2max_ and MAP, consistent with previous studies on elite athletes (Rodríguez et al., 2015). Besides, in highly trained athletes, V̇O_2max_ could be maintained during his/her career and never exceeded the value registered at the time of their first Olympic participation/medal (Lundby and Robach, 2015), showing that other factors (*e.g*., economy/efficiency) are related to endurance performance. Recently, Breda et al. demonstrated an integrated LHTH training model of complex networks and confirmed the prominence of hematological factors, particularly hematocrit and hemoglobin, as central nodes in this adaptive process, suggesting that the LHTH training followed by a period of 15–16 days of re-adaptation to low altitude, is effective in improving the physical performance of paralympic-runners (Breda et al., 2022). Our study shows that hematological parameters ​​increase after an altitude training camp, suggesting that 3 weeks of moderate altitude training are sufficient to promote hematological improvements in elite rowers. However, our results are not associated with increases in maximal aerobic parameters, suggesting that hematological factors could be related to other mechanisms that promote aerobic performance in elite athletes, such as improvements in physiological thresholds and efficiency/economy.

The primary mechanism by which altitude training could induce adaptations related to performance is the increase in the hormone erythropoietin, which stimulates the production of RBC in the bone marrow. Accordingly, there is an increase in hematocrit to facilitate increased oxygen delivery that subsequently could promote an increase in V̇O_2max_. (Levine and Stray-Gundersen, 1997; Levine and Stray-Gundersen, 2005). However, our results showed an increase in hematological parameters without a concomitant increase in V̇O_2max_, showing a decoupling between the increase in oxygen transport capacity and its consumption. A probable explanation is that hypoxia could induce cellular signaling (*e.g*., HIF-1; VEGF), responsible for neovascularization by activating angiogenic mechanisms that mediate skeletal muscle adaptations (Lemieux and Birot, 2021). Besides, training in hypoxia could increase transcript levels of regulators of mitochondrial biogenesis as well as mitochondrial metabolism (Zoll et al., 2006), which could substantially impact training adaptation and exercise performance as physiological thresholds and efficiency/economy. Also, a possible improvement in V̇O_2max_ cannot be discarded once the third adaptation phase has finished. The latter could be induced by the increase in the vascular bed (Padilla et al., 2011) and the number or efficiency of mitochondria in skeletal muscle (Jacobs et al., 2012; Jacobs et al., 2016). This point highlights considering a more prolonged time-course analysis to establish comprehensive kinetics and behaviors of adaptations after completing the intervention (see Limitations section).

As previously proposed, training at altitude induces a series of adaptations that do not depend on the increase in the volume of red blood cells through erythropoiesis or an increase in V̇O_2max_, especially in elite athletes. Instead, they are closely related to responses at the peripheral/molecular level that involve improvements in the economy (*e.g*., mitochondrial efficiency), pH regulation, and muscle buffer capacity (Dubouchaud et al., 2000; Millet et al., 2010). This is consistent with Jacobs et al. (Jacobs et al., 2012), who found that 28 days at a high altitude (3454-m) enhances efficiency in human skeletal muscle mitochondria. However, contrary to the above, Malgoyre et al. (Malgoyre et al., 2021) did not observe significant changes in mitochondrial enzymes linked to efficiency applying a LHTH model; although this valuable study was carried out in female rats, we do not rule out the possibility that it occurs in humans. In addition, the lower supply of oxygen at altitude in conjunction with endurance training induces the activation of mechanisms associated with mitochondrial biogenesis and angiogenesis, mediating skeletal muscle adaptations, promoting the optimization of glucose transport, and an increase in glycolytic enzymes activity (Zoll et al., 2006). Nevertheless, the role those hematological adaptations may have in improving performance cannot be ruled out (*e.g*., blood buffer capacity). Recently, a study showed that the LHTH training model at moderate altitude, followed by a period of 15–16 days in the third phase, is effective in improving the performance of long- and medium-distance paralympic runners (Breda et al., 2022). The authors emphasize that an integrated model of complex networks confirms the importance of hematological parameters, especially hematocrit and hemoglobin, as central nodes in the adaptive process. Thus, while no changes in V̇O_2max_ were apparent in our study, the increase in hematological parameters could be related to other adaptations such as improvements in economy/efficiency and physiological thresholds, explaining the subsequent improvements in sea-level performance after exposure to altitude training.

Power at or related to the second physiological threshold (*e.g*., pVT2 or critical power) are a good indicator of rowing performance (Ingham et al., 2002). Thus, sub-maximal markers of aerobic capacity such as power output at physiological thresholds (*e.g*., ventilatory o lactate threshold) during incremental tests are commonly used variables in rowing, are highly correlated to ergometer performance (Steinacker, 1993; Steinacker et al., 1998), and can be used for planning and monitoring purposes. Furthermore, the power associated with the second physiological threshold (*e.g*., pVT2 and critical power) is sensitive to training (Vanhatalo et al., 2008). An outstanding exercise performance correlates with a high “lactate threshold” (associated with VT1) (Joyner and Coyle, 2008) and critical power (associated with VT2) (Jones et al., 2010), highlighting the importance of our results that show that the LHTH model induced a substantial increase in the power associated with VT1 (7.5%) and VT2 (6.8%). The mechanisms that might cause the shift of pVT1 and pVT2 after LHTH training camp could be related to improvements in gross economy/efficiency, faster V̇O_2_ kinetics, reduced V̇O_2_ slow component, lower cost of breathing, skeletal muscle capillarization, mitochondrial volume density, and/or fiber distribution (Joyner and Coyle, 2008; Lundby and Robach, 2015; van der Zwaard et al., 2021). Also, it is proposed that this improvement might be enhanced by inspiratory muscle training by reducing the V̇O_2_ slow component (Bailey et al., 2010) and the ventilation equivalent for oxygen (V̇E/V̇O_2_), which is a good indication of the economy of respiration. Ventilation of a larger air volume requires a greater activity of the respiratory muscles, which requires a greater percentage of V̇O_2_. This means less oxygen will be available to the skeletal muscles involved during exercise. The more economical the respiratory effort is during exercise, the lower the V̇E/V̇O_2_ ratio (Tharion and Subramani, 2011; Phillips et al., 2020). However, our results showed decreased breathing economy at power VT1, VT2, and V̇O_2max_ (−9.3%, −9.3%, and −2.8%, respectively). These results are concordant with an increase in V̇E at physiological thresholds (8.7% at pVT1 (as % V̇E_max_) and 6.4% at pVT2 (as % V̇E_max_)), despite an improvement in V̇E_max_ (3.3%), suggesting a performance improvement in a breathing-economy independent manner in highly-trained athletes. A possible explanation could be the increases in hematological parameters contributing to buffering changes in the blood (Mairbäurl, 2013) and the improvements founded on economy/efficiency. Finally, increased muscle lactate transport capacity and a higher proportion of type I muscle fibers in trained individuals (Pilegaard et al., 1994) may explain why lactate accumulation (i.e., physiological thresholds) is delayed after training. However, our data do not support this inference because we do not measure lactate values during the tests.

Regarding economy/efficiency, this variable, together with physiological thresholds, is considered an essential determinant of endurance exercise performance (Joyner and Coyle, 2008; di Prampero, 1986), and it is suggested that muscular efficiency and economy might improve with continued endurance training (Joyner and Coyle, 2008). To further highlight the importance of exercise economy/efficiency, some individuals with a relatively modest V̇O_2max_ accompanied by outstanding exercise economy/efficiency can reach elite-athlete status (Lucía et al., 2002; Lucia et al., 2006). Aerobic power consists of three components: (i) V̇O_2max_; (ii) the fraction of maximal uptake that can be sustained during the exercise; and (iii) economy or efficiency of conversion of oxygen consumption into power output (di Prampero, 1986). Thus, changes in endurance performance following adaptation to hypoxia could be due to changes in any of these three components, along with any changes in the contribution of anaerobic power for supramaximal exercise. Improved efficiency with athletic maturity likely involves mechanical and metabolic components, where anthropometry, muscle fiber type, muscle fiber cross-sectional area, and mitochondrial oxidative capacity, among others, could explain exercise efficiency/economy (Joyner and Coyle, 2008; Lundby et al., 2017; van der Zwaard et al., 2021). Rowing efficiency expresses the relationship between energy expenditure and boat velocity, and it depends on the technical skill of the rower (among other variables). Therefore, efficiency could discriminate between rowing and non-rowing athletes. However, there are no differences in efficiency between elite lightweights selected for the World Championships team and those who did not make the team (Lakomy and Lakomy, 1993), suggesting that efficiency on an ergometer is only a rough estimate of technique in the boat (Mäestu et al., 2005). Our results show a substantial decrease in ECR for pVT2 (-5.3%) and a moderate increase in GE% (5.8%), which could imply a performance improvement. Although the causes of the improvement in ECR are not fully understood, a possible cause could be the increases in [Hb] and RBC count, contributing to buffering changes in blood pH by transport of CO_2_ and by binding proton to hemoglobin (Mairbäurl, 2013). Another possible contributing factor is that at least some fast myosin in endurance-trained muscle shifts to a different and perhaps more efficient isoform (Joyner and Coyle, 2008), which could also be mediated by altitude adaptation (Chaillou, 2018). However, our results are not able to confirm the latter hypothesis.

## Limitations

The present study documents a real-world example of LHTH used by elite athletes. However, our study has some limitations that should be considered. First, the lack of a control group is a limitation that must be considered to interpret our findings because we do not know whether the observed improvement could have been achieved through training at sea level alone. Second, to examine the changes in the performance by hypoxia, more time-points after completing the intervention must be considered and thus identify the presence of adaptations induced by altitude, which do not depend on the increase of volume of red blood cells *via* erythropoiesis. However, we conducted the analysis at sea level only 14 days after the altitude camp, hindering the effectiveness of the LHTH training. Finally, the effect of training at altitude on parameters related to sea-level rowing performance on female athletes did not consider the menstrual cycle phases. Future research should consider including this measurement.

## Conclusion

This study is the first to describe the effects of a 3-week moderate altitude training camp (∼2,900 m. a.s.l.) under the LHTH model on performance and economy/efficiency-related variables in elite rowers. The main findings are a rightward shift of the power at ventilatory thresholds, increments in hematological parameters, and improvements in rowing economy and gross efficiency, primarily related to the anaerobic threshold, which is key to improving performance parameters in highly-trained rowers (see Supplementary Figure S1). The results suggest that this strategy is an excellent option to induce positive adaptations related to endurance performance. Future studies should elucidate the mechanisms underlying performance adaptations following this model of altitude training camp.

## Data Availability

The raw data supporting the conclusions of this article will be made available by the authors, without undue reservation.
