# Supplementary material for: Training at moderate altitude improves submaximal but not maximal performance-related parameters in elite rowers
Source: Front Physiol. 2022 Oct 14;13:931325. doi: 10.3389/fphys.2022.931325 (PMC9614325; doi:10.3389/fphys.2022.931325)
Supplement: Supplementary file 1 [file Image1.pdf]

Supplementary Material

Figures

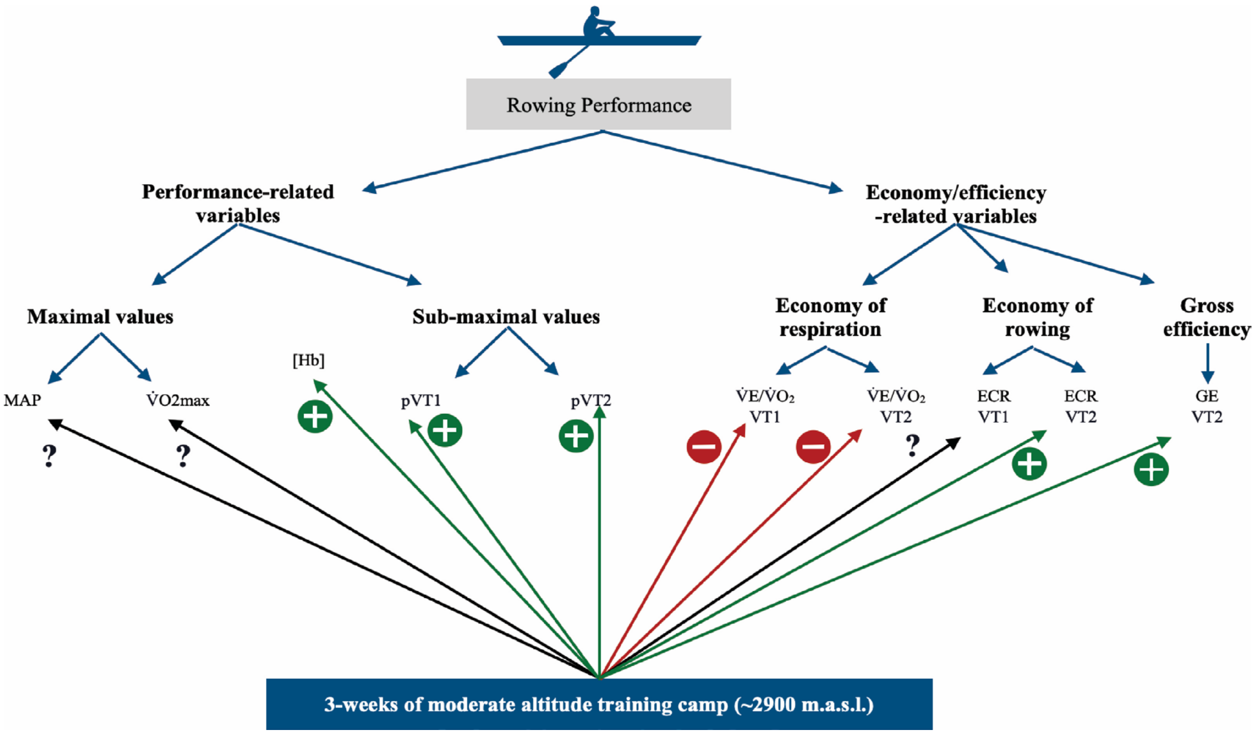

Figure 1. Summary of changes after 3-week moderate altitude training (~ 2900 m.a.s.l.) on parameters related to rowing performance at sea level.
